# Bone loss from Wnt inhibition mitigated by concurrent alendronate therapy

**DOI:** 10.1038/s41413-018-0017-8

**Published:** 2018-05-25

**Authors:** Babita Madan, Mitchell J. McDonald, Gabrielle E. Foxa, Cassandra R. Diegel, Bart O. Williams, David M. Virshup

**Affiliations:** 1 Program in Cancer and Stem Cell Biology, Duke-NUS Medical School, Singapore, 169857 Singapore; 2 Center for Cancer and Cell Biology and Program for Skeletal Disease and Tumor Microenvironment, Van Andel Research Institute, Grand Rapids, MI 49503 USA; 3 Department of Pediatrics, Duke University, Durham, NC USA

## Abstract

Dysregulated Wnt signaling is associated with the pathogenesis of cancers, fibrosis, and vascular diseases. Inhibition of Wnt signaling has shown efficacy in various pre-clinical models of these disorders. One of the key challenges in developing targeted anti-cancer drugs is to balance efficacy with on-target toxicity. Given the crucial role Wnts play in the differentiation of osteoblasts and osteoclasts, acute inhibition of Wnt signaling is likely to affect bone homeostasis. In this study, we evaluated the skeletal effect of small molecule inhibitor of an o-acyl transferase porcupine (PORCN) that prevents Wnt signaling by blocking the secretion of all Wnts. Micro-computed tomography and histomorphometric evaluation revealed that the bones of mice treated with two structurally distinct PORCN inhibitors LGK974 and ETC-1922159 (ETC-159) had loss-of-bone volume and density within 4 weeks of exposure. This decreased bone mass was associated with a significant increase in adipocytes within the bone marrow. Notably, simultaneous administration of a clinically approved anti-resorptive, alendronate, a member of the bisphosphonate family, mitigated loss-of-bone mass seen upon ETC-159 treatment by regulating activity of osteoclasts and blocking accumulation of bone marrow adipocytes. Our results support the addition of bone protective agents when treating patients with PORCN inhibitors. Mitigation of bone toxicity can extend the therapeutic utility of Wnt pathway inhibitors.

## Bone loss: Drug combination reduces toxicity of cancer treatment

Potential bone loss caused by cancer drugs could be mitigated by administering an existing osteoporosis drug. Over-activation of the Wnt signaling pathway, which helps maintain healthy tissues and bone development, is often found in cancer. Scientists are trialing cancer drugs that block a key enzyme PORCN and therefore inhibit Wnt signaling, but these drugs may also adversely affect patients’ bone structure. David Virshup at Duke-NUS Medical School in Singapore and Bart Williams at the Van Andel Research Institute in Michigan, US, and co-workers found that mice treated with PORCN -inhibiting cancer drugs lost bone volume and density within four weeks of exposure. The team then combined the cancer drug with another drug, alendronate, which is already used to treat osteoporosis. This combination targeted aberrant Wnt signaling and limited bone toxicity in the mice.

## Introduction

Wnt signaling plays key roles in many aspects of development and adult tissue homeostasis.^1,2^ WNTs are secreted palmitoleated glycoprotein ligands that bind to cell surface receptors to initiate signaling in diverse tissues and cell types. Downstream signaling commonly leads to stabilization of β-catenin and activation of cell-type specific transcriptional activation.^3^ Mutations that aberrantly activate the Wnt pathway are among the most common events associated with human cancers.^4,5^ Pathological activation of Wnt signaling is also critical in the progression of various fibrotic disorders.^6,7^ Targeted therapies to inhibit the Wnt pathway are being developed and have shown efficacy in various pre-clinical models of Wnt addicted cancers and in fibrosis (reviewed in ref. ^8^). For example, the secretion of all Wnts can be blocked by inhibition of PORCN. PORCN is an endoplasmic reticulum resident O-acyl transferase that catalyzes the palmitoleation of all Wnts.^2,9^ Pre-clinical studies of PORCN inhibitors have demonstrated robust activity against several tumor types. Orally available drugs that inhibit PORCN have been developed and have progressed to human clinical trials.^6,10–13^ However, concerns regarding mechanism-based toxicity of Wnt pathway inhibitors could impact the clinical utility of these agents.

Multiple studies have demonstrated an important role for Wnt/β-catenin signaling in bone formation, maintenance, and accrual during different stages of the life (reviewed in refs. ^14,15^). Early in development, Wnt/β-catenin signaling is required for the differentiation of mesenchymal precursor cells to osteoblasts. Conditional genetic ablation of β-catenin or simultaneous deletion of Wnt co-receptors *Lrp5* and *Lrp6* in the mesenchymal progenitors or osteoblast-committed precursors blocks their differentiation and maturation to osteoblasts, leading to their absence in membranous bones.^16–19^ In the absence of β-catenin these osteochondro-progenitors differentiate into chondrocytes. Conversely, ectopic activation of stabilized β-catenin leads to enhanced ossification and suppression of chondrocyte formation.^16,19^ In addition to differentiation, Wnt/β-catenin signaling also coordinates bone acquisition by regulating the activity of both osteoclasts and osteoblasts. In vitro, osteoblasts lacking β-catenin exhibit impaired maturation and mineralization with elevated expression of osteoclast differentiation factor ligand (RANKL), and diminished expression of the RANKL decoy receptor, osteoprotegerin.^20^ Thus, inactivation of β-catenin in mature osteoblasts promotes osteoclast differentiation hence enhanced bone resorption.^21^ Conditional loss-of-Wntless, a chaperone required for secretion of Wnts from osteoblasts also severely impairs bone formation and inhibits the accrual of bone mass within the first 3 weeks after birth. There is dramatic reduction of the cortical and trabecular bone mass in these mice emphasizing the crucial role of osteoblast secreted Wnt ligands in bone mass accrual.^22^

Compared to the osteoblasts, osteocytes are long-lived and constitute 90% of the bone cells. Ectopic activation of canonical Wnt signaling by expressing dominant active β-catenin in the osteocytes results in an increase in the trabecular bone volume and bone mineral density of both the axial and appendicular skeleton.^23^ Conversely, loss-of-β-catenin in the mature osteoblasts and/or osteocytes causes severe osteopenia associated with increased osteoclastogenesis.^20^ These findings demonstrate that in adults Wnt/β-catenin signaling promotes the ability of differentiated osteoblasts to inhibit osteoclast differentiation.

In this study, we analyzed the effect of PORCN inhibitors that block all Wnt secretion on bone structure and homeostasis in mice. We further examined if simultaneous administration of a clinically approved anti-resorptive, alendronate, could mitigate loss-of-bone mass and extend the beneficial anti-tumor effects of PORCN inhibitors to a larger number of human patients.

## Results

### Exposure to PORCN inhibitors results in significant bone loss

**Fig. 1:**
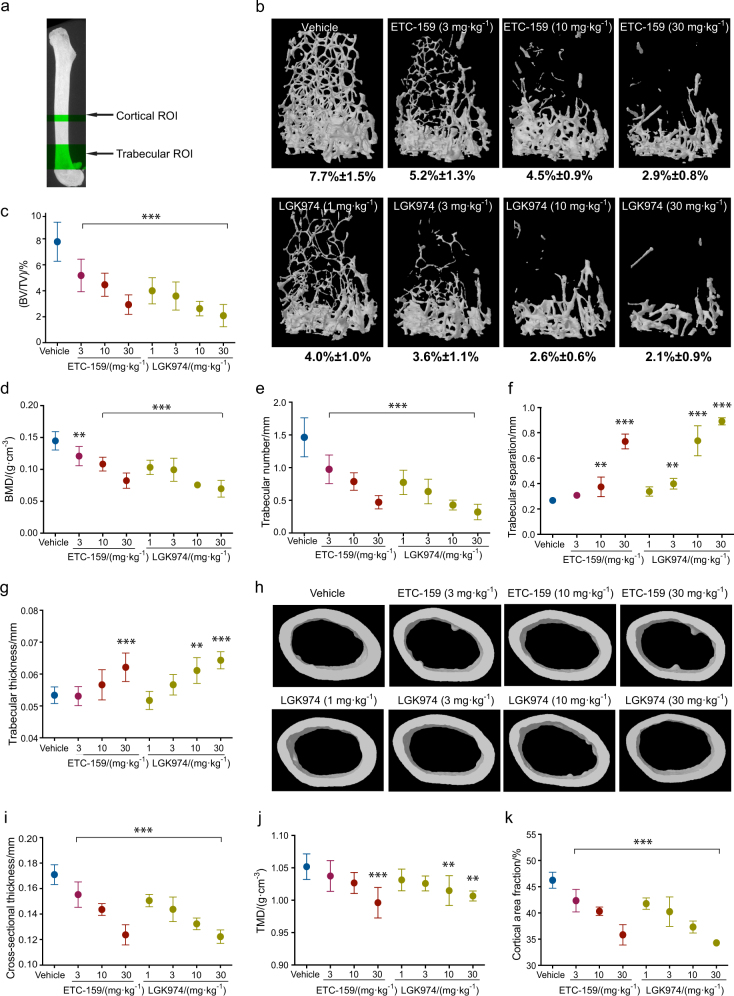
Wnt secretion inhibitors reduce bone volume and bone mineral density in dose-dependent manner: C57/BL6 mice were treated daily for 4 weeks with vehicle or the indicated oral doses of ETC-159 or LGK974. Femurs were then collected and analyzed by µCT. **a** Schematic of regions analyzed by µCT. µCT parameters assessed were **b**, **c** trabecular bone volume fraction (bone volume (BV)/tissue volume (TV)), **d** bone mineral density (BMD), **e** trabecular number, **f** trabecular separation, and **g** trabecular thickness. **h** Representative cross-sectional images from the cortical region. µCT parameters assessed were **i** average cross-sectional thickness, **j** tissue mineral density (TMD), and **k** cortical area fraction (CAF = bone area (B.Ar)/tissue area (T.Ar)). Data are presented as mean ± SD, (*n* = 6 mice/group). The significance of changes compared to the Vehicle group is indicated. *P ≤ 0.05, **P ≤ 0.01, ***P ≤ 0.001.

To analyze the effect of Wnt secretion inhibitors on bone homeostasis, 8-week-old female mice were treated with two structurally distinct PORCN inhibitors, ETC-159 and LGK974.^10,12^ After 4 weeks of continuous treatment, the bones were collected and the trabecular and cortical regions of the left femurs were analyzed by micro-computed tomography (µCT) (Fig. 1a). ETC-159 and LGK974 produced comparable effects, including reduction in total bone volume and bone mineral density (Fig. 1b–d, h–k). Dose-dependent effects on bone volumes were demonstrated with 3–30 mg·kg^-1^ ETC-159 and 1–30 mg·kg^-1^ LGK974. These dose ranges were based on their pharmacokinetic properties and their effective dose in pre-clinical mouse models.^10,12,13^ A comprehensive trabecular analysis revealed a decrease in bone mineral density (BMD) and trabecular number (Fig. 1b–e) while the trabecular separation and thickness increased (Fig. 1f–g). There was also a slight reduction in the tissue mineral density in the cortical region, as well as reductions in total cortical area fraction and cortical thickness with either ETC-159 or LGK974 treatment (Fig. 1h–k). Compared with the trabecular regions, the effect of PORCN inhibitors in the cortical region was milder.

### Alternate-day dosing has the same effect on bone mass as daily administration

**Fig. 2:**
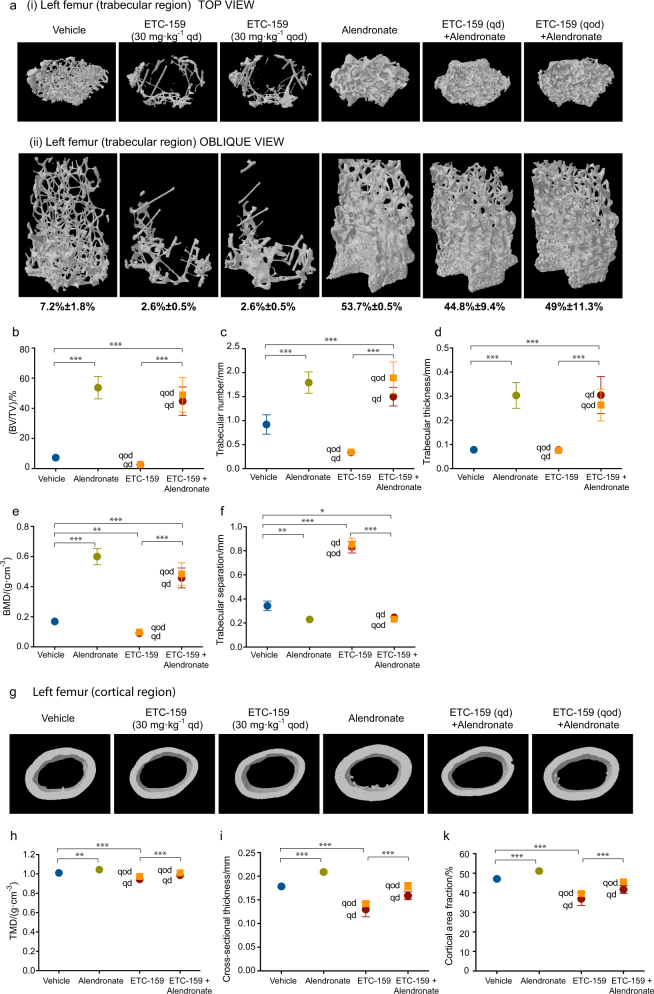
Alendronate treatment restores bone density in ETC-159 treated mice: C57/BL6 mice were treated for 8 weeks as indicated with vehicle, alendronate (1 mg·kg^-1^ qd, subcutaneously), or ETC-159 (30 mg·kg^-1^ qd or qod, orally) alone or in combination with alendronate. Femurs were then collected and analyzed by µCT. **a** Representative images of the epiphyseal region from (i) top and (ii) oblique views. The parameters quantitated using µCT are **b** trabecular bone volume (BV)/tissue volume (TV), **c** trabecular number, **d** trabecular thickness **e** bone mineral density (BMD) and **f** trabecular separation. **g** Representative top view images of the cortical region of the femur. The parameters quantitated using µCT are **h** tissue mineral density (TMD), **i** cross-sectional thickness and **d** cortical area fraction (CAF = B.Ar/T.Ar). Yellow squares (qod dose); red circles (qd dose). Data are presented as mean ± SD, (*n* = 9 mice/group). *P ≤ 0.05, **P ≤ 0.01, ***P ≤ 0.001.

We evaluated if changes in the dosing schedule of ETC-159 could mitigate its side effects on bone volume and bone mineral density. We previously found that ETC-159 has a half-life of <2 h in mice.^10^ Thus, we hypothesized that an every other day (qod), rather than a daily (qd), dosing schedule might reduce treatment-induced bone loss. However, there was no difference in multiple parameters of bone mass and framework in mice treated every other day relative to those treated daily (Fig. 2a–f).

### Alendronate treatment can overcome ETC-159-induced bone loss

Bisphosphonates are commonly used to treat patients suffering from osteoporosis. We tested whether simultaneous treatment with the clinically approved bisphosphonate, alendronate, can overcome the effect of Wnt secretion inhibitors on bones. Mice were administered 1 mg·kg^-1^ alendronate daily along with ETC-159 treatment. As expected, μCT analysis revealed alendronate alone significantly increased trabecular bone volume fraction (BV/TV), trabecular number, trabecular thickness, and bone mineral density (BMD) that was accompanied by a decrease in trabecular separation (Fig. 2a–f). Importantly, co-administration of alendronate with ETC-159 mitigated the bone loss seen with ETC-159 alone. The measured bone parameters were comparable to the mice treated with alendronate alone (Fig. 2a–f). Cortical μCT analysis showed similar results when alendronate was administered. Treatment with alendronate alone and in combination with ETC-159 significantly increased tissue mineral density (TMD), cross-sectional thickness, and cortical area fraction (Fig. 2g–j).

**Fig. 3:**
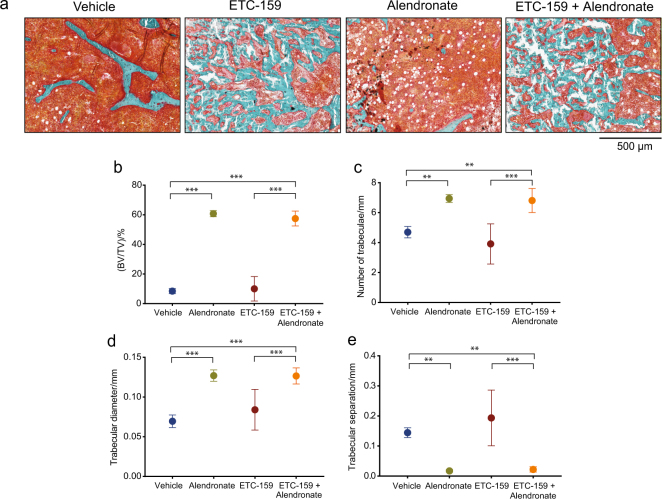
Effects of ETC-159 and alendronate on static bone histomorphometry. **a** Brightfield images of trichrome stained femoral sections from the indicated groups. Static histomorphometry of sagittal sections measured. **b** Bone volume (BV)/tissue volume (TV) **c** trabecular number, **d** trabecular diameter, **e** trabecular separation. Mice per group was vehicle (*n* = 5), alendronate (*n* = 3), ETC-159 (*n* = 5), and ETC-159 and alendronate, (*n* = 6). The data represent the mean ± S.D. **P ≤ 0.01, ***P ≤ 0.001.

Histomorphometric analysis confirmed our analyses with μCT as there was a significant increase in trabecular bone volume fraction BV/TV, trabecular number, trabecular diameter, and a decrease in trabecular separation with treatment of alendronate alone and in conjunction with ETC-159 (Fig. 3a–e).

**Fig. 4:**
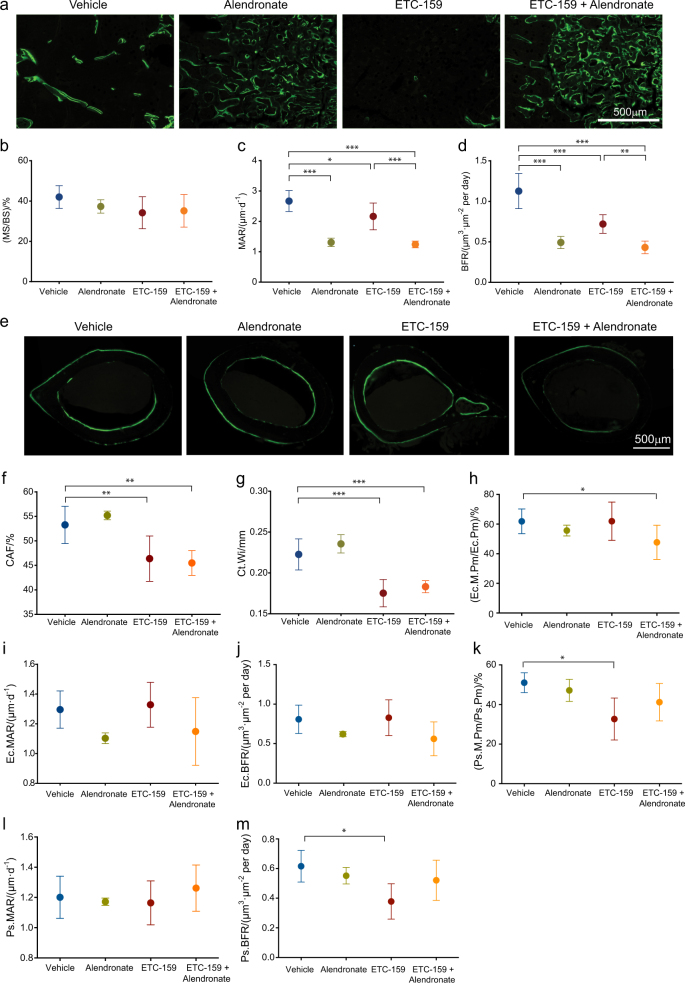
Regulation of bone formation rate by alendronate in ETC-159 treated mice: **a** Representative fluorescence microscopy images of calcein labeled trabecular bone from treatment groups as indicated. Dynamic histomorphometry of trabecular bone using sagittal sections assessed **b** mineralizing surface (MS) /bone surface (BS), **c** mineral apposition rate (MAR) and **d** bone formation rate (BFR). **e** Representative fluorescence microscopy images of calcein labeled cortical bone sections from indicated groups. Dynamic histomorphometry of sagittal sections measured **f** Cortical area fraction (CAF) **g** cortical width **h** endocortical-mineralizing perimeter (Ec.M.Pm)/endocortical perimeter (E.Pm), **i** endocortical mineral apposition rate (Ec.MAR), **j** endocortical bone formation rate (Ec.BFR), **k** periosteal mineralizing perimeter (Ps.M.Pm)/periosteal perimeter (Ps.Pm), **l** periosteal mineral apposition rate (Ps.MAR) and **m** periosteal bone formation rate (Ps.BFR). *n* = 5 or 6 mice per group. The data represent the mean ± SD *P ≤ 0.05, **P ≤ 0.01, ***P ≤ 0.001.

The effects of alendronate co-administration were further examined by dynamic histomorphometry following calcein injection (Fig. 4a, e). The mineralization surface (MS/BS) of trabecular bone did not change between treatment groups (Fig. 4b). However, treatment with alendronate and ETC-159 alone and in combination resulted in a significant decrease in mineral apposition rate (MAR) and consequently a significant decrease in bone formation rate (Fig. 4c, d).

Dynamic histomorphometric analysis of cortical bone (Fig. 4e) showed a significant decrease in cortical area fraction (CAF) and cortical width with ETC-159 treatment; however, the increase in CAF and cortical width with addition of alendronate was not significant (Fig. 4f–g). Dual treatment with ETC-159 and alendronate significantly decreased endocortical-mineralizing perimeter (Ec.M.Pm/Ec.Pm) when compared with vehicle control (Fig. 4h). None of the treatments caused a significant change in endocortical MAR or BFR (Fig. 4i, j). Periosteal mineralizing perimeter (Ps.M.Pm/Ps.Pm) and BFR significantly decreased with the addition of ETC-159 alone (Fig. 4k, l) but could be restored on co-treatment with alendronate. The periosteal MAR and BFR were not significantly affected with any treatment (Fig. 4l, m). These analyses suggest that alendronate is acting as an anti-resorptive to increase amounts of trabecular and cortical bone. Taken together, these data indicate that alendronate treatment considerably improves overall bone mass and mitigates the bone loss effects of PORCN inhibitor.

### ETC-159 and alendronate treatment influence bone framework

**Fig. 5:**
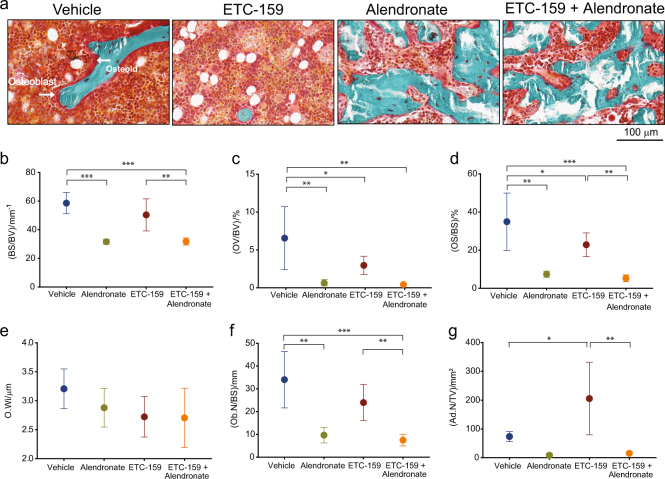
Effects of ETC-159 and alendronate on static bone histomorphometry. **a** Brightfield images of trichrome stained femoral sections from the indicated groups. Static histomorphometry of sagittal sections measured **b** bone surface (BS)/bone volume (BV), **c** osteoid volume (OV)/bone volume (BV), **d** osteoid surface (OS)/bone surface(BS), **e** osteoid width (O.Wi), **f** number of osteoblasts (Ob.N)/bone surface(BS), and **g** number of adipocytes(Ad.N)/tissue volume(TV). Mice per group was vehicle (*n* = 5), alendronate (*n* = 3), ETC-159 (*n* = 5), and ETC-159 and alendronate, (*n* = 6). The data represent the mean ± SD *P ≤ 0.05, **P ≤ 0.01, ***P ≤ 0.001.

To determine the changes in bone development patterns, bone structure and cellular differences were analyzed by Goldner’s trichome staining (Fig. 5a). The trabecular BV/TV changes driven by ETC-159 and alendronate treatment (Fig. 3b) were followed by structural changes. Bone surface area (BS/BV) was moderately reduced from 58.6 mm^−1^ in the vehicle to 31.6 mm^−1^ following treatment with ETC-159 alone (Fig. 5b). However, treatment with alendronate alone or concurrently with ETC-159 significantly decreased BS/BV, indicating complex trabecular structures reverted to more simple spherical structures (Fig. 5b). This alendronate-driven decrease in structural complexity was coupled with increased trabecular number and diameter, and was not affected by PORCN inhibition (Fig. 3c–e). Consistent with the increase in trabecular diameter and number, trabecular separation significantly decreased in all mice receiving alendronate, regardless if they were also exposed to ETC-159 (Fig. 3e).

Consistent with the observation made with calcein staining that the bone formation rate was reduced, osteoid volume (OV/BV) decreased when mice were treated with ETC-159, and further decreased on treatment with alendronate alone or in combination ETC-159. The osteoid volume decreased from 6.5% to 2.98% in ETC-159 treated mice and to 0.64% in alendronate-treated mice. This dropped to 0.43% in dually treated mice (Fig. 5c). There was a similar pattern of change in the osteoid surface in the ETC-159 and alendronate-treated mice (Fig. 5d). Although, ETC-159 to a lesser extent, and alendronate to a greater extent resulted in reduction of osteoid volume and surface, the change in osteoid width between the treatment groups was not significant (Fig. 5e). Decrease in the osteoid volume and osteoid surface upon PORCN inhibition and alendronate treatment could be attributed to reduction in the number of osteoblasts that are responsible for secretion of the bone matrix (Fig. 5f).

One notable change in overall bone makeup was a significant increase in adipocytes when mice were treated with ETC-159. The number of adipocytes increased from 73.6 per mm^2^  in vehicle-treated mice to 205 per mm^2^ in ETC-159-treated mice (Fig. 5g). However, the addition of alendronate to ETC-159 reverted the adipocyte phenotype back to normal. The observed effect of increased adipogenesis on inhibition of Wnt signaling is consistent with the role of Wnt signaling in regulating the balance between adipocytes and osteoblasts during mesenchymal stem cell differentiation (reviewed in ref. ^24^).

### Response of osteoclasts to alendronate and ETC-159 treatment

**Fig. 6:**
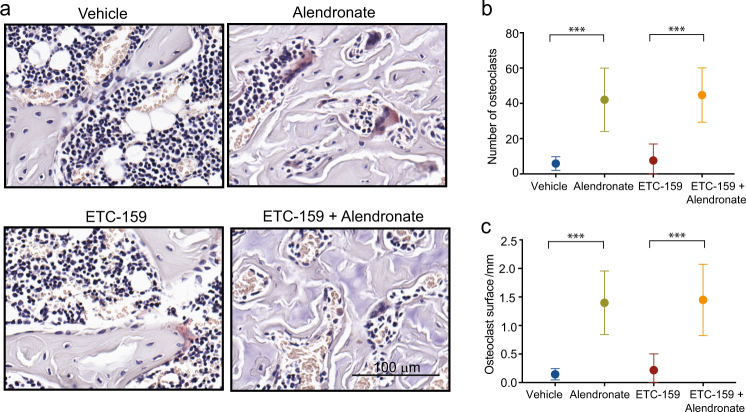
Response of osteoclasts to alendronate and ETC-159 Treatment. **a** Brightfield images of TRAP stained sagittal sections from vehicle, alendronate, ETC-159, and dually treated groups. Osteoclast quantification included **b** average number of osteoclasts per sample and **c** average osteoclast surface per sample. *n* = 5 or 6 mice per group. The data represent the mean ± SD ***P ≤ 0.001.

Due to the substantial loss-of-bone in mice treated with PORCN inhibitors, we analyzed the osteoclasts by TRAP staining (Fig. 6a–c). Treatment with ETC-159 alone did not have a significant effect on the number or size of osteoclasts. Alendronate led to significant changes in osteoclast numbers and surface, irrespective of the co-administration of ETC-159. The number of osteoclasts increased from an average of 5.83 per sample in vehicle-treated mice to 42.0 in mice treated with alendronate alone, and remained at 44.7 in dual-treated mice (Fig. 6b). Similarly, osteoclast surface also increased from 0.145 mm in vehicle-treated mice to 1.4 mm in alendronate-treated mice and 1.45 mm in dually treated mice (Fig. 6c). ETC-159 alone did not have a significant effect on osteoclast surface (Fig. 6c). These alterations in osteoclasts are consistent with previous studies demonstrating increases in the number and size of osteoclasts upon alendronate treatment, perhaps reflecting the fact that osteoclasts are undergoing protracted apoptosis.^25^

### Serum parameters of resorption and formation upon alendronate and ETC-159 exposure

**Fig. 7:**
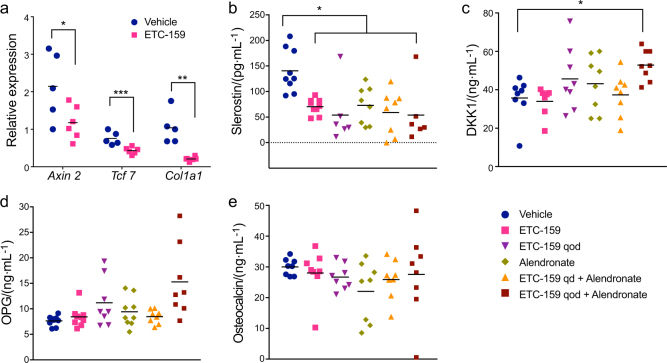
Assessing Wnt target genes and serum markers of osteoclast and osteoblast activity. **a** Relative expression of Wnt target genes in the bones of mice treated orally with ETC-159 as assessed by qRT-PCR. Levels of **b** Sclerostin **c** DKK1, **d** and osteoprogeretin. **e** Osteocalcin in the serum of mice as measured using Luminex based ELISA assay. *P ≤ 0.05.

Treatment with ETC-159 reduced expression of Wnt target genes *Axin2*, *Tcf7* and *Col1a1* in the bone, indicating that systemic Wnt pathway inhibition blocks Wnt signaling in bone that then leads to changes in bone density and volume (Fig. 7a). Finally, we also assessed if systemic Wnt inhibition altered serum biomarkers of osteoclast and osteoblast activity. PORCN inhibition led to a significant decrease in serum concentration of the Wnt inhibitor sclerostin, with a trend to a stronger suppression with qod dosing (Fig. 7b). This may reflect a response to decreasing local and global Wnt signaling. This decrease in sclerostin was not reversed by concurrent alendronate administration. A small increase in DKK1 concentration, also a negative regulator of Wnt signaling, was observed with qod dosing of ETC-159, which was enhanced with concurrent treatment with alendronate (Fig. 7c). However, the concentration of osteoblastogenesis markers, osteoprotegerin (OPG) and osteocalcin had non-significant changes (Fig. 7d, e). This is consistent with alendronate specifically inhibiting the activity of osteoclastic enzymes without affecting the activity of existing osteocytes.

## Discussion

Activation of Wnt/β-catenin signaling is one of the more common events associated with the development of human cancer. This has led to an intense focus on developing agents that inhibit this pathway as candidate therapeutics for cancer treatment. One approach has been to target the activity of PORCN, an enzyme that catalyzes the addition of a palmitoleic acid to a conserved serine residue in all mammalian Wnts. This lipidation is required both for proper secretion of Wnts, as well as their binding to Frizzled family members to activate downstream signaling cascades.^26–28^ Several different PORCN inhibitors have been developed and show promising results in pre-clinical studies. These are in various stages of clinical development.^10–12^

Other therapies designed to inhibit Wnt signaling are also in clinical development. One example is vantictumab (OMP18R5), which binds to Frizzled 1, 2, 5, 7, and 8 and blocks the ability of Wnt ligand to activate signaling via these receptors. The Phase I clinical trials of vantictumab were temporarily halted due to “on-target” bone adverse events. The potentially deleterious effects of therapies targeting Wnt signaling on the skeleton was not unexpected, given the role of Wnt signaling in regulating homeostasis of the skeleton.^19^ For example, loss-of-function mutations in the Wnt co-receptor LRP5 result in osteoporosis pseudoglioma syndrome that is associated with extremely low bone mass at an early age.^29^ Conversely, point mutations in LRP5 that prevent its inhibition by extracellular negative regulators of the pathway, such as DKK1 are associated with high-bone mass.^30^ Similarly, loss or severe reductions in the expression of sclerostin, an extracellular inhibitor of Wnt signaling increases bone mass. In fact, these observations provided the foundation for the development of romosozumab, an anti-sclerostin antibody, which has shown promising results in a Phase 3 trial to treat osteoporosis.^30^

Our finding that systemic administration of PORCN inhibitors increases bone marrow adiposity is consistent with earlier studies showing that alterations in Wnt signaling alter the balance between osteoblasts and adipocytes. Wnt signaling, likely driven by Wnt6, Wnt10a, or Wnt10b acting via Frizzleds 1, 2, or 5 and Lrp5/6,^31^ restricts adipocyte differentiation and stimulates osteogenesis. In addition, PORCN inhibition was previously shown to block the anti-adipogenic and pro-osteoblastic effects of another agent used for cancer treatment, 5-Aza-dC,^32^ suggesting that adipogenic induction at the expense of osteoblastogenesis is a common effect of treatment with these agents.

Alendronate, like other members of the bisphosphonates, is typically thought to maintain bone mass via its ability to block the activity of osteoclasts. Our results here suggest that it also can act to influence the differentiation of the osteoblast lineage in vivo. Whether this is a result of direct influence on mesenchymal stem cell differentiation or an indirect consequence of its effects on osteoclasts will be the subject of future work. It is important to note that previous studies have found that alendronate can inhibit adipogenesis and stimulate osteoblastogenesis in several in vitro studies.^33^ In addition, future work will explore whether other bisphosphonates or other agents that interfere with osteoclast activation such as Denosumab will also be beneficial in the context of PORCN inhibition.

In summary, we demonstrate here that PORCN inhibition results in acute loss-of-bone that can be mitigated by the co-administration of alendronate. Given that alendronate was FDA approved in 1995 and has been used by thousands of women to treat osteoporosis, our work should be rapidly translatable to work in human clinical trials to allow patients to benefit from the efficacy of PORCN inhibitors in treating tumors dependent on Wnt ligands for growth and maintenance while reducing the side effects on the skeleton.

## Material and methods

### Mice

C57/Bl6 mice 8–10 weeks old were purchased from InVivos Singapore. All the experiments were performed in compliance with the guiding principles and were approved by the Duke-NUS Medical School “Institutional Animal Care and Use Committee.” Mice were orally administered ETC-159 formulated in 50% PEG400 at a dosing volume of 10 μL·g^-1^ body weight. Alendronate was administered subcutaneously.

### Bone microcomputed tomography

Hind limbs from killed animals were fixed in neutral buffered formalin for 48 h at room temperature followed by storage in 70% ethanol. Right femurs were isolated for analyses via microcomputed tomography (µCT) (SkyScan1172, Bruker). At the time of scanning, femurs were wrapped in phosphate buffered saline (PBS) soaked gauze and placed within a tube containing PBS. To quantitatively compare the effects of two different small molecule PORCN inhibitors (ETC-159 and LGK974) at varying concentrations, femurs were scanned with an X-ray energy of 50 kV, X-ray intensity of 200 μA, at an isotropic voxel size of 5.99 µm^3^ (Fig. 1). To quantitatively assess the effects of ETC-159 and/or alendronate treatment on bone, whole femurs were scanned with an X-ray energy of 50 keV, X-ray intensity of 200 µA, at an isotropic voxel size of 13.3 µm^3^ (Fig. 2b–f, h–j). For high-resolution qualitative images, representative samples from each treatment group were scanned at an isotropic voxel size of 3.19 µm^3^ (Fig. 2a, g). The resulting three-dimensional cross-sectional images are shown. Trabecular bone was analyzed in the distal epiphysis, with a volume of interest (VOI) beginning 0.25 mm proximal to the growth plate and spanning 2.5 mm proximally. The region of interest (ROI) was drawn manually a few pixels inside of the endosteal surface. Cortical bone was analyzed in the distal metaphysis, with a VOI beginning 55% of the overall bone length from the proximal tip of the femur and extending distally 0.6 mm (Fig. 1a). Analyses were performed with the CTAn analysis software package.

### Dynamic histomorphometry

The mice were intraperitoneally injected with 10 mg·kg^-1^ calcein on 8 and 3 days prior to killing. Femurs were fixed in formalin for 48 h at room temperature followed by storage in 70% ethanol prior to embedding and sectioning. Neutral buffered formalin fixed femurs were dehydrated in graded ethanol solutions ranging from 70% to 90%. The samples were cleared using xylene and embedded in methylmethacrylate (MMA). Cortical wafers 30–50 μm thick were cut just distal to the midshaft using a Extech Labcut 150, and sanded by hand. The femurs were re-embedded in MMA for sagittal sectioning. Sagittal sections were cut 5 μm thick, at the distal end of the femur, using a Tungsten Carbide Profile D-Profile Microtome Blade. Sections were mounted, coverslipped, and imaged using a fluorescent microscope at ×20 magnification. All cortical bone parameters were measured using Bioquant Osteo v14.1.60 software, and trabecular bone parameters were measured using Bioquant Osteo v16.1.6 software. Bone area (B.Ar), tissue area (T.Ar), cortical width (Ct.Wi) periosteal (Ps), endocortical (Ec), and trabecular (Tb) bone formation constituents were calculated by measuring unlabeled perimeter, single-labeled surface (sLS), double-labeled surface (dLS) and the inter-label width (Ir.L.Wi). Derived indices were calculated in Bioquant Osteo v14.1.60 and Bioquant v16.1.6. using the standard histomorphometric formulas, which include: cortical area fraction [CAF = B.Ar/T.Ar (%)] mineralizing surface over bone surface [MS/BS = (dL.Pm+(sL.Pm/2)/Pm) (%)], mineral apposition rate [MAR = (Ir.L.Wi/5 days) (μm·d^-1^)], and bone formation rate [BFR/BS = MAR*(MS/BS) (μm^3^·​μm^-2^ per day)].

### Static histomorphometry

The sagittal sections were mounted and stained with Goldner’s Trichrome stain using the manufacturer’s protocol. The sections were imaged using an Aperio ScanScope slide scanner at ×20 magnification. Trabecular bone parameters were calculated using Bioquant Osteo v16.1.6 software. The region of interest drawn for each sample was ~1 mm in length, with all borders 100 μm away from the growth plate and any cortical bone. Within the region of interest, tissue volume (TV), bone volume (BV), bone surface (BS), trabecular number (Tb.N), trabecular diameter (Tb.Dm), trabecular separation (Tb.Sp), osteoid volume (OV), osteoid surface (OS), osteoid width (O.Wi), number of adipocytes (N.Ad), and number of osteoblasts (N.Ob) were measured. Derived indices were calculated with Bioquant v16.1.6. using the standard histomorphometric formulas, which include: bone volume over tissue volume [BV/TV (%)], bone surface over bone volume [BS/BV (%)], osteoid surface over bone surface [OS/BS (%)], osteoid volume over bone volume [OV/BV (%)], number of osteoblasts over bone surface [(Ob.N/BS)/mm], and number of adipocytes over total volume [Ad.N/TV/mm^2^].

To investigate osteoclast parameters, the remaining NBF fixed femurs from each mouse were decalcified in 10% EDTA. The samples were cleared in graded ethanol, followed by xylene and embedded in paraffin. Sagittal sections were cut 5 μm thick, at the distal end of the femur, using a Tungsten Carbide Profile D-Profile Microtome Blade. The sections were mounted and stained for tartrate-resistant acid phosphatase (TRAP) using Napthol AS-BI phosphate (Sigma) as the substrate and Pararosaniline Hydrochloride (Sigma) and Sodium Nitrite (Fisher) for the chromogenic reaction. The sections were imaged using an Aperio ScanScope slide scanner at ×20 magnification. Number of osteoclasts (N.Oc) and osteoclast surface (Oc.S) were measured using Bioquant v16.1.6 software.

### RNA isolation and qRT-PCR

Mice were treated orally with 30 mg·kg^-1^ ETC-159 daily for 7 days. The bones were collected and homogenized in Trizol (Thermo Fischer Scientific). Total RNA was isolated using manufacturer’s protocol and reverse transcribed with iScript reverse transcriptase (BioRAD). Real time quantitative PCR (qPCR) was performed with SsoFast^™^ EvaGreen^®^ assay from BioRad. ACTB was used as housekeeping gene.

### Statistical analysis

For histomorphometric and µCT parameters, Welch’s *T*-test with a Benjamini–Hochberg false discovery rate correction was used to determine significant differences between measurements or derived indices within each treatment group.

### Serum enzyme-linked immunosorbent assay

Serum was collected from the animals at study termination (8 weeks of treatment). Levels of OPG, Osteocalcin, SOST, and DKK1 were measured using Milliplex MagPlex kit according to manufacturer’s specifications (Millipore, Darmstadt, Germany).
